# Sunflower-Like Nanostructure with Built-In Hotspots for Alpha-Fetoprotein Detection

**DOI:** 10.3390/molecules26041197

**Published:** 2021-02-23

**Authors:** Xiaoyu Zhao, Aonan Zhu, Yaxin Wang, Yongjun Zhang, Xiaolong Zhang

**Affiliations:** 1 School of Material and Environmental Engineering, Hangzhou Dianzi University, Hangzhou 310018, China; zhaoxy@hdu.edu.cn (X.Z.); Wangyaxin1010@126.com (Y.W.); 2 College of Chemistry, Nankai University, Tianjin 300071, China; aonanzhu@126.com; 3 College of Physics, Jilin Normal University, Changchun 130103, China; zhangxiaolong10086@126.com

**Keywords:** LSPR, SERS, alpha-fetoprotein

## Abstract

In the present study, a sunflower-like nanostructure array composed of a series of synaptic nanoparticles and nanospheres was manufactured through an efficient and low-cost colloidal lithography technique. The primary electromagnetic field contribution generated by the synaptic nanoparticles of the surface array structures was also determined by a finite-difference time-domain software to simulate the hotspots. This structure exhibited high repeatability and excellent sensitivity; hence, it was used as a surface-enhanced Raman spectroscopy (SERS) active substrate to achieve a rapid detection of ultra-low concentrations of Alpha-fetoprotein (AFP). This study demonstrates the design of a plasmonic structure with strong electromagnetic coupling, which can be used for the rapid detection of AFP concentration in clinical medicine.

## 1. Introduction

Nowadays, non-communicable diseases, such as cardiovascular diseases, cancer, and diabetes, have increased the mortality rate worldwide. For instance, China is positioned first globally in terms of the morbidity and mortality rate. The statistics of cancer patients in China are as follows: there is one cancer patient out of every 65 people, more than 4 million people are diagnosed with cancer every year, more than 10,000 people are diagnosed with cancer every day, and more than five people die of cancer every minute. In particular, the mortality rate of liver cancer patients is incredibly high. Therefore, early screening and diagnosis of liver cancer patients is crucial. At present, the detection methods of hepatocellular carcinoma mainly include computed tomography, magnetic resonance imaging, and histological methods [1,2,3]. However, these detection techniques have high costs as they rely on expensive equipment and are not suitable for early patient screening. Therefore, the development of a cost-effective, fast, efficient, and reliable detection method is highly warranted.

In 1927, the Indian physicist Raman first discovered the Raman spectrum. Raman spectroscopy has a special light scattering effect that occurs when light interacts with a matter. When a beam of light strikes a medium, most of the light is either absorbed, transmitted, or reflected, and the remainder gets scattered by the medium. Similarly, in the process of photon scattering, the wavelength (or frequency) of most of the scattered light does not change, which is called Rayleigh scattering [4,5,6]. Rayleigh scattering is also known as elastic scattering as it is a result of an elastic collision between the incident photon and the scattering center. In contrast, when a very small part of the scattered light shows changes in the wavelength (or frequency), it is called Raman scattering. The Raman spectrum is closely related to the wiggle of atoms in matter and the wiggle and vibration of chemical bonds, while the vibration mode of different groups of atoms is unique and produces a specific Raman spectrum. Based on the fingerprint effects, the Raman spectrum can also be used to analyze and identify solid, liquid, and gas samples [6,7,8,9]. With the advancement of 40 years, SERS spectrum has finally evolved into a high-sensitivity detection method with strong spectral characteristics and is not susceptible to solution influence. It has been widely applied in chemistry, material science, analysis science, surface science, biomedicine, and other research fields [10,11,12,13,14]. Compared with the traditional detection method, SERS spectral detection technology has the advantages of the super high sensitivity of single molecule detection and reflection of the intrinsic fingerprint information of substances.

Therefore, we chose SERS spectral detection technology for the detection of liver cancer cell markers. At present, Alpha-fetoprotein (AFP), as a common marker, is widely used in the diagnosis of liver cancer patients. To date, several SERS active substrates have been reported, including gold nanoparticles [15,16,17], silver nanoparticles [18], and Fe_3_O_4_@Ag composite nanoparticles [19]. However, these SERS substrates have limited practical applications due to their poor sensitivity, poor selectivity, and poor stability. In order to overcome these limitations, we designed a sunflower-like nanostructure coupled with a strong electromagnetic field that showed higher SERS enhancement and better signal reproducibility. Here, we reported that this is a candidate for an excellent SERS active substrate, which is annotated as a sunflower-like structure. In addition, the sunflower-like structure has achieved the detection of ultra-low-concentration liver cancer cell marker AFP, which has further improved the practical application development of SERS spectroscopy detection technology.

## 2. Material and Methods

### 2.1. Materials

The solid 4-mercaptobenzoic acid (4-MBA) was purchased from Sigma-Aldrich Co., Ltd. The phosphate-buffered saline (PBS; 0.01 M, pH 7.4) and bovine serum albumin (BSA) were obtained from Beijing Yinuokai Technology Co., Ltd. The AFP-L3 enzyme-linked immunoassay kits were purchased from Beijing Dingguo Changsheng Biotechnology Co., Ltd. Polystyrene microsphere with a diameter of 200 nm was purchased from Duke Co., Ltd. A solution consisting of 10 wt.% aqueous solution and Au (99.999%) targets were obtained from Beijing TIANQI Advanced Materials Co., Ltd. (HZTQ), whereas the deionized water (18.2 MΩ·cm^−1^) was obtained from a Millipore water purification system. The Si wafer (1 × 1 cm^2^), used as a substrate, was cleaned with ethanol and deionized water followed by plasma cleaning (Fischione model 1020) for 5 min.

### 2.2. Modification of SERS Active Substrate

The sunflower-like structures were immersed in a 10^−3^ mol/L 4-MBA ethanol solution for 30 min and washed thoroughly three times to eliminate the unabsorbed 4-MBA molecules. Then, they were incubated in 10 mL of mixed solution of NHS/EDC (EDC 0.2 mM, NHS 0.5 mM, volume rate: 5:1) for 120 min, then cleaned three times with deionized water and dried in a nitrogen environment. The SERS active substrates further modify the Anti-AFP species (20 μL anti-AFP, 37 °C, 240 min). The samples were also dried under the protection of nitrogen. Then, we used PBS instead of serum for AFP dilution, using the BSA as a serum-like medium. Later, the AFP antigen with different concentrations was modified on the SERS active substrate. We then took a further measurement of the SERS spectrum after drying in a nitrogen environment.

### 2.3. Finite-Difference Time-Domain (FDTD) Simulations

The finite-difference time-domain (FDTD) software was employed to calculate the hotspot distribution pattern of the nanostructure in a theoretical field. To confirm the relationship between the theoretical and experimental values, the physical model parameters used in the FDTD numerical calculation were obtained with reference to the experimental results. Two sources polarized along the orthogonal axes were used as the excitation light. Their relative amplitudes and phases were adjusted to obtain an x-polarized plane wave with its phase set to 0° and a y-polarized plane wave with its phase set to 90°. According to the periodicity of the structure, different boundary conditions were selected in different directions. The periodic boundary and perfectly matched layer boundary conditions were applied to the x–y plane and z direction, respectively. The grid accuracy was set to 2 nm due to the limited availability of computer resources. The auto-shutoff minimum was fixed to 1 × 10^−5^, and the overall simulation time to 1000 s. The simulation temperature was set to 300 K. The fitting parameters of the silver material were obtained from the material library of the FDTD.

### 2.4. Characterization

A series of scanning electron microscopy (SEM) images were obtained using a JEOL 6500F field emission scanning electron microscope with a primary electron energy of 15 kV. A Renishaw Raman system model 2000 confocal microscopy spectrometer equipped with a charge-coupled device (CCD) detector and a holographic notch filter was used to acquire the Raman spectra of the samples. The model of the magnetron control sputtering system was ATC 1800-F, USA AJA.

## 3. Results and Discussion

### 3.1. Construction and Characterization of the Sunflower-Like Nanostructure

**Figure 1:**
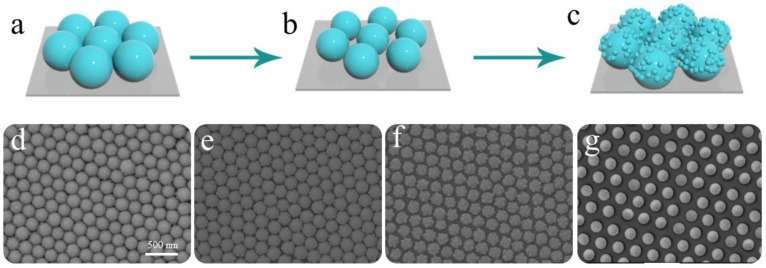
(**a**–**c**) Schematic diagram of the preparation phases of sunflower-like structure; (**d**–**g**) SEM image of the samples with different etching time (3, 4, 5, and 6 min, respectively) after 20 min of deposition.

The preparation process of the sunflower-like structure could be divided into three stages. The first stage consisted of the preparation of a close-packed single-layer polystyrene spheres array on the silicon wafer substrate at the gas–liquid interface by using the self-assembly method [20] (Figure 1a). As depicted in Figure 1b, ion etching was performed on the polystyrene spheres array during the second stage to obtain a template array with different spacing and the polystyrene spheres with the increase of etching time. In the third stage, the sunflower-like structure was obtained by a magnetron sputtering system to deposit gold material on the etched array template (Figure 1c).

The SEM image of the preparation process of the sunflower-like structure is displayed in Figure 1d–g. To develop SERS active substrates coupled with a strong electromagnetic field, we selected the array template after different etching times and sputtered Au material. Identical sputtering conditions were adopted during the magnetron sputtering process. Figure 1d corresponds to the morphology of the Au material deposited after the etching of the sample for 3 min, which shows a relatively smooth surface. As depicted in Figure 1e, an alternation in the etching time increased the spacing of the spheres, and the surface of spheres developed the synaptic nanoparticles. As expected, Figure 1f shows more synaptic nanoparticles on the surface of the spheres. The synaptic nanoparticles provided abundant hotspots on the surface of the array structures. When the spacing of the spheres was further increased, the synaptic nanoparticles began to disappear, which was not conducive for the development of hotspots (Figure 1g). As the etching time increases, the size of the microspheres in the template decreases rapidly, resulting in the loss of the convex structure on the surface of the microspheres. Therefore, the array structure with a strong hotspot distribution was prepared under the etching time of 5 min (Figure 1f).

**Figure 2:**
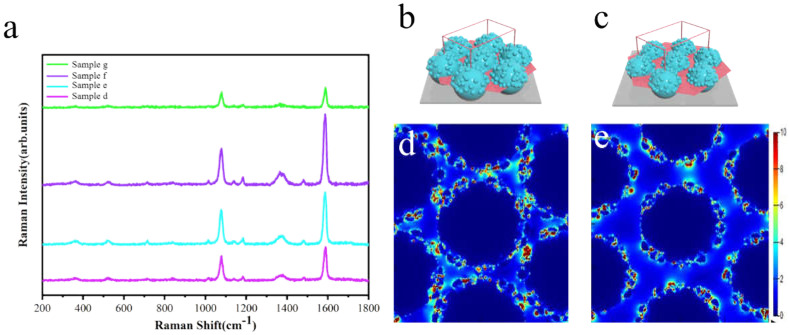
(**a**) SERS spectra of the 4-MBA probe molecules absorbed onto sample d–g. (**b**,**c**) Ideal morphology of the structures used in the FDTD simulation: the light slices represent the locations of the frequency-domain field monitor. (**d**,**e**) Cross-section of the local electric field distribution of the corresponding structures.

To evaluate the potential effect of the sunflower-like structure in SERS-active substrates, a series of SERS signals generated by 4-MBA (10^−3^ M) probe molecules were allowed to absorb on sample d–g (corresponding with Figure 1d–g). The spectra were measured with a Renishaw Raman system by using HeCd laser irradiation (633 nm). The typical exposure time for each measurement was 10 s with a one-time accumulation. The laser power attenuation was set to 1%. The intensity of the SERS signals confirmed that the sunflower-like structure has an excellent SERS enhancement performance. Furthermore, we employed the FDTD software to calculate the hotspot distribution of the sunflower-like structure and to prove the contribution of the synaptic nanoparticles on the sphere surface for hotspot development. Figure 2b,c illustrates the physical model used for simulating and locating the field monitor. The minimum simulated period is represented by the area of the wireframe, whereas the light slices represent the locations of the frequency domain field monitor, which are located on the x–y plane. Similarly, Figure 2d,e depicts the calculation results. These calculation results helped us to find the hotspots that were mainly distributed around the synaptic nanoparticles. Additionally, when the unit structure distance is too large, it could weaken the coupling between the synaptic nanoparticles. Therefore, the sunflower-like structure with the synaptic nanoparticles and suitable unit spacing was considered suitable as SERS active substrates.

### 3.2. Detection of AFP with Sunflower-Like Structure SERS Active Chips

**Figure 3:**
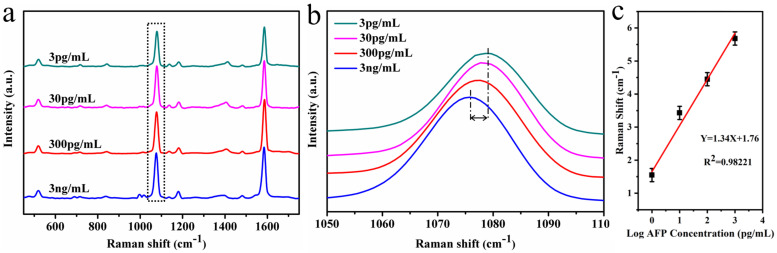
(**a**) SERS spectrum of an active subtrate decorated with different concentrations of AFP antigen. (**b**) A magnified image of the marked area in the dotted line in Figure 3a. (**c**) Function plot of the AFP concentration logarithm and the 1075 cm^−1^ peak frequency shift.

The sunflower-like structure was selected as the SERS active substrate for detecting AFP due to its excellent SERS enhanced performance. Firstly, 4-MBA probe molecules were modified on the SERS active substrate surface. A total of 10 mL of EDC-NHS mixed solution was used for activation of the carboxyl group to induce the generation of the MBA-derived coupling agent [21,22]. Afterward, the anti-AFP species could be attached to the SERS active substrate via the MBA-derived coupling agent. Finally, the SERS active chips were immersed in different concentrations of AFP solution for specific identification, and measurements were taken of their SERS spectrum (Figure 3a). Accumulating studies have reported that the frequency shift of the peak at 1075 cm^−1^ could be used as a standard for quantitative analysis of AFP concentration changes [23]. The peak at 1075 cm^−1^ belongs to the ph-S-Au vibration mode, which is attributed mainly to the quantitative changes in anti-AFP/4-MBA and AFP/anti-AFP/4-MBA interactions [24,25,26]. The magnified SERS signals from the probe molecules showed significant frequency shifts of the peak at 1075 cm^−1^, as AFP concentrations increased from 0.003 to 3 ng/mL. Figure 3b shows a larger version of the box selection in Figure 3a. For quantitative analysis, we plotted the logarithm function of the AFP concentration and the frequency shift of the peak at 1075 cm^−1^ (Figure 3c) according to the equation Y = 1.34 X + 1.76, where Y is the change in the frequency shift and X is the logarithmic concentration of AFP (R^2^ is the correlation coefficient). This revealed that sunflower-like structures have excellent properties for detecting AFP even at very low concentrations.

## 4. Conclusions

The present study demonstrates the preparation, characterization, and application of sunflower-like structure. The development of numerous hotspots in the synaptic nanoparticles on the sphere surface was confirmed by FDTD simulation. SERS was used as an active chip for detecting the liver cancer cell marker AFP due to its excellent performance. This technique enhanced the detection limit of AFP to a range of 0.003 to 3 ng/mL. This study provides an insight for the early detection of the chest packet of liver cancer.

## Data Availability

No new data were created or analyzed in this study. Data sharing is not applicable to this article.

## Sample Availability

Samples of the compounds are not available from the authors.
